# Kyste hydatique du foie compliqué d'un accident vasculaire cérébral ischémique: à propos d'un cas

**DOI:** 10.11604/pamj.2015.22.355.8108

**Published:** 2015-12-11

**Authors:** Olfa Turki, Mabrouk Bahloul, Kamilia Chtara, Kais Regaieg, Sondes Haddar, Mounir Bouaziz

**Affiliations:** 1 Service de Réanimation Médicale, CHU Habib Bourguiba, Sfax, Tunisie; 2 Service d'Imagerie Médicale CHU Habib Bourguiba, Sfax, Tunisie

**Keywords:** Kyste hydatique du foie, choc anaphylactique, accident vasculaire cérébral ischémique, Hydatid cyst of the liver, anaphylactic shock, ischemic stroke

## Abstract

Le kyste hydatique du foie (KHF) est une maladie assez répandue dans les pays nord-africains. La rupture post-traumatique ou spontanée du kyste compliquée d'un choc anaphylactique et d'un AVC ischémique a été exceptionnellement rapportée. Nous rapportons un cas d'un kyste hydatique du foie (KHF) fissuré et compliqué d'un choc anaphylactique et d'un AVC ischémique.

## Introduction

Le kyste hydatique du foie (KHF) est une maladie endémique dans les pays du Maghreb [1–3]. La rupture post-traumatique ou spontanée du kyste compliquée d'un choc anaphylactique et d'un AVC ischémique a été exceptionnellement rapportée [1]. Nous rapportons un cas de Le kyste hydatique du foie (KHF) fissuré et compliqué d'un choc anaphylactique et d'un AVC ischémique.

## Patient et observation

Nous rapportons le cas d'un patient Z.F âgé de 27 ans (originaire de Kasserine), sans antécédents pathologiques notables admis au service de réanimation pour prise en charge d'une altération de l’état de conscience avec état de choc.

L'interrogatoire de la famille révèle une notion de conflit juste avant le début de la symptomatologie. A l'admission, le patient avait un score de Glasgow à 3/15 sans signes de localisations, des pupilles normodilatés, une bradypnée à 10 c/min et une saturation pulsée à 89% sous 12L/min d'oxygène. La pression artérielle était imprenable et le pouls à 150 battements par minutes, avec des extrémités froides. Un remplissage vasculaire par un litre de cristalloïdes a été réalisé et le patient intubé et ventilé à vif (sans sédation, ni curare). I l a bénéficié d'une tomodensimètrie cérébrale devant la symptomatologie neurologique initiale qui était sans anomalies. L'hypothèse d'intoxication aigue a été avancée et un lavage gastrique a été réalisé et a été non productif (n'est pas en faveur d'une intoxication). Trente minutes après son admission enréanimation, l'examen clinique a montré l'apparition de plaques d'urticaire au niveau du tronc associée à un état de choc anaphylactique. Le patient a reçu une titration d'adrénaline (100ug) et une corticothérapie a base d'hémisuccinate d′hydrocortisone (100 mg en intraveineux direct). Devant la persistance de l’état de choc, le patient a été mis sous catécholamine à base d'adrénaline à la seringue électrique. Les examens biologiques pratiqués à l'admission ont montré une hyperleucocytose à 23100/mm^3^ avec une hyper-éosinophilie à 2%, un taux d'hémoglobine à 12.1g/dl et un taux des plaquettes à 83000 élts/mm3. Le bilan hépatique a montré une cytolyse hépatique (ASAT: 376 UI/L et ALAT: 294 UI/L). Le bilan rénal a montré une insuffisance rénale avec une urée à 27 mmol/l et un taux de créatinine à 495 µmol/l). Le reste du bilan a monté une acidose métabolique sévère (pH: 7.09; HCO3: 12 mmol/l) avec un trou anionique augmenté à 25 mmol/l.

**Figure 1:**
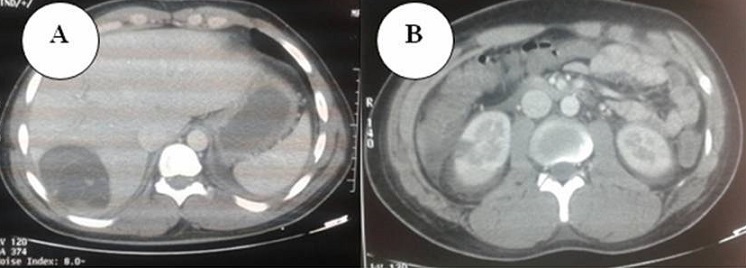
(A) TDM abdominale montrant la présence d'un kyste hydatique du foie fissuré; (B) la présence de plages hypodenses corticaux rénaux bilatéraux d'origine très probablement ischémique (B)

**Figure 2:**
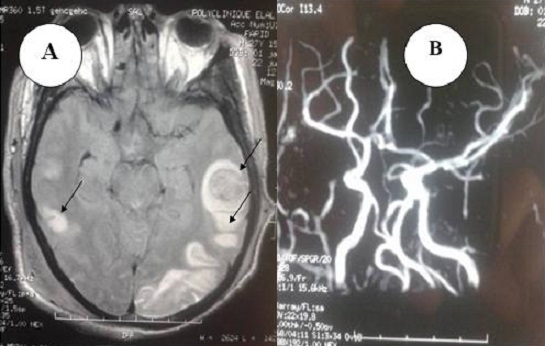
(A) IRM cérébrale mettant en évidence des lésions anoxo-ischémiques; (B) angio-IRM d'aspect normal

Devant la détresse respiratoire et les perturbations du bilan biologique (rénale et hépatique), un scanner thoraco-abdominal a été réalisée et n'a pas objectivé d'embolie pulmonaire. Cependant, les coupes abdominales hautes ont montré la présence d'un kyste hydatique du segment 7 du foie mesurant 77mm de grand axe au contact de la veine sus-hépatique (Figure 1), et on a noté la présence de plages hypodenses corticaux rénaux bilatéraux d'origine très probablement ischémique (Figure 2). Une échographie abdominale a été réalisée et a montré une formation kystique cloisonnée du segment VII de 7 x 6 cm à membrane décollé, en contact avec la veine sus hépatique droite qui reste perméable. Cet aspect est en faveur d'une fissuration du kyste hydatique. La sérologie hydatique a été positive au test immonologique de western blot avec présence d'une bande spécifique de 7 kdalton. Le diagnostic retenu est celui d'un choc anaphylactique secondaire à une fissuration d'un kyste hydatique du foie. Le dosage des IgE a montré un taux supérieur à 1000 UI/mL (pour un taux normal < 150 UI/mL) confirmant l'origine immuno-allergique de l’état de choc initial. L’évolution a été marquée par l'aggravation de la fonction rénale (urée à 35 mmol/l et une créatinémie à 557 µmol/l) avec installation d'une oligoanurie nécessitant le recours à plusieurs séances d'hémodialyse. Cependant, nous n'avons pas noté des stigmates biologiques d'insuffisance hépato-cellulaire. Sur le plan neurologique, l’évolution a été marquée par l'absence de réveil malgré l'arrêt de sédation depuis 72h. Une IRM cérébrale a été demandée et qui a montré des lésions anoxo-ischémiques au niveau des noyaux gris centraux et du splénium du corps calleux associés des lésions cortico-sous corticales temporo-occipitales gauche et temporo-parietale droites et frontales bilatéraux d'allures ischémiques avec transformation hémorragique (Figure 2).

L'angio-IRM a été normale et elle a éliminé une cause vasculaire. Devant ce tableau le diagnostic d'un AVC ischémique secondaire à la fissuration du kyste hydatique a été évoqué. Une échographie des troncs supra-aortique a été réalisée et était sans anomalies. Une Angio TDM cérebrale a été faite 20 jours plutard, elle a montré l'absence de thromboses veineuses et artérielles avec une stabilité des lésions. Secondairement et quelques jours après le rétablissement d'un état hémodynamique stable, la fonction rénale est devenue normale. Le patient a été trachéotomisé pour une cause neurologique (absence de réveil et un score de Glasgow à 10/15). Il a été sevré de la ventilation mécanique à j15 d’évolution puis il a été mis sortant à domicile. Un examen clinique de contrôle a été réalisé 1 mois après sa sortie et a montré une récupération neurologique totale.

## Discussion

L'hydatidose est une parasitose cosmopolite endémique en Tunisie. Elle peut se développer dans n'importe quel organe en particulier au niveau du foie et du poumon [1–5]. La localisation hépatique est la plus fréquente (2/3 des cas) et est expliquée par le passage portal obligatoire du parasite [4]. Le traitement repose essentiellement sur la chirurgie. Celle-ci peut être accompagnée de complications certes rares mais graves pouvant engager le pronostic vital. Les réactions allergiques peuvent se manifester par un choc anaphylactique qui se caractérise par sa brutalité de survenue ainsi que sa gravité. L’état de choc anaphylactique est une complication rare rapportée souvent au cours de la chirurgie du kyste hydatique [5]. En dehors des circonstances peropératoires, quelques cas sporadiques de KHF compliqué d’état de choc anaphylactique ont été décrits dans la littérature [1, 5].

La rupture post-traumatique ou spontanée du kyste est une complication rare survenant suite à une augmentation de la pression intra-kystique [1, 2]. Dans notre cas, l'interrogatoire de la famille révèle une notion de conflit juste avant le début de la symptomatologie faisant évoquer la possibilité de l'une de ces deux hypothèses.

Dans notre cas, la nature anaphylactique de l’état de choc a été retenue devant l'installation brutale de l'hypotension, l'apparition de plaques d'urticaire au niveau du tronc associée avec une hyper éosinophilie et d'un taux très élevé des IgE à la biologie. Le diagnostic de la fissuration du kyste a été confirmée par les données radiologiques (échographie abdominale, TDM abdominale), biologique (sérologie hydatique positive) et le taux des IgE très élevé. Le mécanisme de ces réactions anaphylactiques dues à la libération du liquide hydatique est complexe. Les deux hypothèses physiopathologiques avancées dans la littérature sont celle d'une réaction d'hypersensibilité de type (I) liée à une libération massive des immunoglobulines E [1, 5] et la réaction anaphylactique ou anaphylactoïde secondaire à une activation du complément avec libération d'anaphylatoxines [1, 5]. Notre observation supporte le premier mécanisme. En effet le taux des IgE était très augmenté chez notre patient.

L'AVC ischémique est une présentation inhabituelle du KHF rompu. Dans la littérature, des cas d'AVC ont été décrits avec la localisation cardiaque du kyste [1, 5], mais exceptionnellement avec la localisation hépatique [1]. La survenue chez notre patient d'un AVC ischémique pourrait être expliquée par plusieurs mécanismes. La première hypothèse est la migration d'un embole hydatique qui va traverser le filtre pulmonaire, vu la plasticité de l'embryon hexacanthe ce qui lui confère la possibilité de passer partout où peut passer une hématie [1]. La localisation cérébrale serait alors en rapport avec la présence d'un foramen ovale patent. La deuxième hypothèse est une vascularite cérébrale secondaire à la libération de produits inflammatoires et vasoactifs suite à la fissuration du kyste [1, 4, 5]. Mais la brutalité du tableau n'est pas en faveur de ce diagnostic qui est souvent d’évolution insidieuse. La troisième hypothèse est le syndrome de vasoconstriction cérébrale réversible (SVCR) ou pseudovascularite [1, 2]. Cette affection est due à une anomalie transitoire et réversible de la régulation du tonus artériel cérébrale, qui entraîne une vasoconstriction et une vasodilatation multifocale et diffuse [6]. La régression des signes cliniques peut cadrer avec le tableau de SVCR. Dans notre cas, l'hypothèse de la migration d'un embole hydatique qui va traverser le filtre pulmonaire et/ou le syndrome de vasoconstriction cérébrale réversible semblent être les plus probables. En effet, la brutalité de l'installation et la multiplicité des lésions ischémiques à l'IRM cérébrale et la présence de lésions d'ischémie rénales représentent des arguments en faveur de ces deux hypothèses physiopathologiques. Cependant, les résultats de l'angioscanner cérebral réalisé 20 jours plutard (l'absence de thromboses veineuses et artérielles avec une stabilité des lésions ischémique) représente un argument supplémentaire en faveur de la migration d'un embole hydatique responsable d'une obstruction artérielle.

## Conclusion

Le développement d'un choc anaphylactique et d'un AVC ischémique dans un pays endémique doit inciter à la recherche d'une rupture post-traumatique ou spontanée d'un kyste hydatique du foie. Ainsi, le kyste hydatique fissuré du foie doit être ajoutée à la liste étiologique des AVC ischémique chez les sujets jeunes.
